# Advances in Biomarkers and Endogenous Regulation of Breast Cancer Stem Cells

**DOI:** 10.3390/cells11192941

**Published:** 2022-09-20

**Authors:** Wenmin Chen, Lu Zhang, Suling Liu, Ceshi Chen

**Affiliations:** 1 Key Laboratory of Animal Models and Human Disease Mechanisms, Kunming Institute of Zoology, Chinese Academy of Sciences and Yunnan Province, Kunming 650201, China; 2 Kunming College of Life Sciences, The University of the Chinese Academy of Sciences, Kunming 650201, China; 3 Fudan University Shanghai Cancer Center & Institutes of Biomedical Sciences, State Key Laboratory of Genetic Engineering, Cancer Institutes, Key Laboratory of Breast Cancer in Shanghai, The Shanghai Key Laboratory of Medical Epigenetics, Shanghai Key Laboratory of Radiation Oncology, The International Co-laboratory of Medical Epigenetics and Metabolism, Ministry of Science and Technology; Shanghai Medical College, Fudan University, Shanghai 200032, China; 4 Jiangsu Key Lab of Cancer Biomarkers, Prevention and Treatment, Collaborative Innovation Center for Cancer Medicine, Nanjing Medical University, Nanjing 211166, China; 5 Academy of Biomedical Engineering, Kunming Medical University, Kunming 650500, China; 6 The Third Affiliated Hospital, Kunming Medical University, Kunming 650118, China

**Keywords:** BCSC, biomarker, signal pathway

## Abstract

Breast cancer is one of the most common cancers. Even if breast cancer patients initially respond to treatment, developed resistance can lead to a poor prognosis. Cancer stem cells (CSCs) are a group of undifferentiated cells with self-renewal and multipotent differentiation characteristics. Existing evidence has shown that CSCs are one of the determinants that contribute to the heterogeneity of primary tumors. The emergence of CSCs causes tumor recurrence, metastasis, and therapeutic resistance. Previous studies indicated that different stemness-associated surface markers can identify other breast cancer stem cell (BCSC) subpopulations. Deciphering the critical signaling networks that are involved in the induction and maintenance of stemness is essential to develop novel BCSC-targeting strategies. In this review, we reviewed the biomarkers of BCSCs, critical regulators of BCSCs, and the signaling networks that regulate the stemness of BCSCs.

## 1. Introduction

Cancer has become one of the world’s public health problems. According to the latest statistics, there are 19.3 million new cancer cases worldwide, and 2.26 million new cases of breast cancer among them, therefore breast cancer is ranked first [1]. In China, according to cancer statistics in 2018, it was found that the incidence of breast cancer is increasing, and the onset age is becoming younger [2]. Research suggests that breast cancer is closely related to genetic and environmental factors [3,4]. Breast cancer is a highly heterogeneous disease, divided into four subtypes, including Luminal A, Luminal B, HER2 positive, and triple-negative breast cancer (TNBC) [5].

Despite the rapid development of medical technology, breast cancer is still a recurrence risk. BCSCs are often associated with recurrence, metastasis, therapeutic resistance, and other biological behaviors [6]. Emerging evidence indicates that, although BCSCs account for only a small subset of tumor cells, they have tumorigenic potential, multipotent differentiation, and self-renewal capabilities [7]. BCSCs are derived from various sources, including normal stem-cell-gained mutations and de-differentiation of cancer cells induced by microenvironment components [8].

Targeting BCSCs seems to be an effective way to improve breast cancer treatment. The more we know about BCSCs, the more we understand how to eliminate them. In this review, we summarized the latest research progress on BCSCs, focusing on the identification biomarker of BCSCs and signal pathways regulating their stemness.

## 2. Identification of BCSCs

Since the concept of CSCs was proposed, scientists have tried identifying and isolating CSCs in various cancers. In breast cancer, BCSCs were initially identified as the CD24^−^/CD44^+^ phenotype. As few as 100 CD24^−^/CD44^+^ BCSCs isolated from breast cancer tissues of patients were able to form tumors in immunocompromised mice [9]. In vitro studies further verified that the CD24^−^/CD44^+^ cancer cell population exhibited a self-renewal capacity and could differentiate into bulk cancer cells [10]. Although cell surface markers confer specificity to identify and isolate BCSCs, scientists have also tried to utilize stem-associated characteristics to define stem cells. Aldehyde dehydrogenases (ALDHs) are enzymes responsible for aldehydes’ oxidation (dehydrogenation). Ginestier et al. discovered that the phenotype and function of cells with ALDH hyperactivity were like those of breast cancer stem cells. They used the ALDEFLUOR assay to identify and isolate cells with high ALDH enzyme activity (ALDH^+^ cells). The efficiency of the mammosphere formation of ALDH^+^ cells was approximately 13%, while no mammospheres formed from ALDH^−^ cells [11]. ALDH^+^ and CD24^−^/CD44^+^ cell populations partially overlap and are interchangeable. Subsequently, Mollie et al. found that some of the cell populations isolated from a BRCA1-deficient mouse mammary tumor were composed of a low percentage of CD24^−^/CD44^+^ cells and harbored 2–5.9% CD133^+^ cells. These CD133^+^ breast cancer cells exhibited strong sphere formation, tumor initiation, and drug resistance capabilities, suggesting that CD133 was another BCSC marker. Notably, the observation that CD133^+^ BCSCs did not overlap with CD24^−^/CD44^+^ BCSCs raised the hypothesis that there might be heterogeneity in BCSCs [12].

Several new membrane proteins and prognostic markers have been reported to regulate stemness in specific subtypes of breast cancer, such as protein C receptor (PROCR) [13], tetraspanin 8 (TSPAN8) [14], tumor endothelial marker 8 (TEM8) [15], epsilon sarcoglycan (SGCE) [16], and Ki-67 [17]. PROCR, as a surface marker on mammary stem cells, was first identified as being expressed on multipotent mammary stem cells located in the basal layer of the mammary gland [18]. PROCR-positive cells exhibited a high self-renewal capacity in transplantation assays and was differentiated into multiple lineages, as indicated by lineage tracing. In breast cancer, interfering with PROCR expression significantly decreased the number of CSCs, and dampened tumor growth and recurrence in TNBC [13,19]. More research, such as lineage tracing, is needed to elucidate the characteristics of these newly identified BCSC subpopulations. Ki-67, an established prognostic indicator for assessing malignancy, was reported as being required to maintain the cancer stem cell niche in breast cancer [17]. Equivalent or higher levels of Ki-67 were observed in the metastatic lesion, indicating Ki-67 might be strongly associated with metastatic potential. However, the knockout of Ki-67 in the breast epithelia cell line MCF10A did not decrease cell proliferation [17]. The development and fertility of the Ki-67-knockout mice were normal [20].

Another way to identify CSCs is based on their features of biological behaviors, such as employing Hoechst 33,342 staining, or using a PKH26-based label retention assay to discriminate stem cells. The Hoechst 33,342 dye is a cell-permeant fluorescent stain used to label double-stranded DNA. Although all types of cells take up Hoechst, stem cells efflux faster than other cells. Based on this, cells in the side area of the Hoechst-positive cells were gated as stem cells using fluorescence-activated cell sorting (FACS) analysis and named side population (SP). SP cells not only express high levels of stemness genes but are also resistant to chemotherapy and radiotherapy. SP cells have been isolated in several cancers, especially those without surface markers [21]. PKH26, as a cell membrane dye, is mainly used to study the approach of cell division. Because PKH26 is equally inherited by daughter cells, the intensity of PKH26 reflects the number of cell divisions. Usually, stem cells or CSCs divide much more slowly and retain more of the PKH26 dye in daughter cells than normal cells or cancer cells. Thus, it is convenient to isolate or track these stem cells in a quiescent state in vivo using the PKH26 staining assay.

## 3. Endogenous Factors That Regulate BCSCs

### 3.1. Epigenetic Factors

BCSCs have a unique gene expression signature, predominantly determined by an array of epigenetic and transcription factors. B lymphoma Mo-MLV insertion region 1 homolog (BMI1) is a polycomb-group (PcG) transcriptional repressor that is well documented in promoting BCSC self-renewal and tumorigenicity [22]. The molecular chaperone heat shock protein 90 alpha (HSP90α) can maintain the expression of BMI1, subsequently increasing the self-renewal ability of BCSCs [23]. In contrast, miR-494-3p inhibits BCSC self-renewal by targeting BMI1 [24]. Recently, the activated interleukin-1 receptor type 2 (IL1R2) was shown to recruit USP15 in order to stabilize BMI1 and promote the self-renewal and metastasis of BCSCs [25].

Zeste homolog 2 (EZH2) is the catalytic subunit of polycomb repressor complex 2 (PRC2), which methylates histone H3 lysine 27 (H3K27) to inhibit transcription, and enhances the mammosphere-forming ability of BCSCs [26]. EZH2 promotes the self-renewal of BCSCs and increases the percentage of BCSCs by activating the Wnt pathway [27,28]. Li et al. discovered that the protein arginine methyltransferase 1 (PRMT1) catalyzes the asymmetric methylation of EZH2 to foster breast cancer proliferation, metastasis, and tumorigenesis [29,30].

Lysine-specific demethylase 1 (LSD1) selectively acts on histone H3K4 and H3K9 through a yellow flavin adenine dinucleotide (FAD)-dependent oxidative reaction. LSD1 has the effect of dual-transcriptional activators and inhibitors [31,32]. LSD1 may indirectly modulate CSCs via cancer-associated fibroblasts (CAFs) and the tumor microenvironment [32].

PRMT1 is an asymmetric arginine N-methyltransferase in mammalian cells that catalyzes the asymmetric demethylation of histone H4 arginine 3 (H4R3) and modifies the active chromatin [33]. In addition, PRMT1 modulates cell function through the methylation of forkhead box O1 (FOXO1) and ERα proteins [34]. PRMT1 can induce MCF10A cells to develop stem cell characteristics and self-renewal abilities [33]. PRMT1 may increase the attributes of TNBC stem cells by activating the signal transducer and activator of transcription 3 (STAT3). Moreover, the epidermal growth factor receptor (EGFR) signal mediated by PRMT1 may contribute to the upregulation of ZEB1 and the promotion of BCSC generation [34]. The same family member PRMT5 is a type II methyltransferase that controls the symmetrical demethylation of arginine residues on target proteins in the cytoplasm and nucleus. It modulates transcription through the methylation of transcription factors, such as nuclear factor kappa B (NF-κB), p53, and E2F Transcription Factor 1 (E2F1) [35]. PRMT5 promotes the expansion of stem cells through histone methylation and the expression of forkhead box P1 (FOXP1) and Kruppel-like Factor 4 (KLF4), thereby enabling the development of breast tumors and chemotherapy resistance [36]. Similarly, PRMT5 methylates KLF5 to prevent its phosphorylation, ubiquitination, degradation, and facilitates the transcription of downstream target genes, thereby promoting the maintenance and proliferation of BCSCs [37].

Epigenetic-factor histone deacetylases (histone deacetylases, HDACs) target lysine residues to facilitate chromatin condensation and to regulate biological processes such as mitosis, differentiation, autophagy, and apoptosis [38,39]. HDAC1, HDAC5, and HDAC7 are necessary to maintain BCSCs. HDAC7 overexpression is sufficient to increase the CSC phenotype, manifested in the increases in sphere formation and tumor-initiating cell frequency [39]. HDAC5 silencing inhibits the growth, migration, and invasion of BCSCs and increases apoptosis [38]. HDAC1 stabilizes KLF5 protein by preventing its ubiquitination and degradation [40]. The expression of HDAC1 and KLF5 is positively associated with breast cancer [40]. HDAC inhibitors inhibit the expression of KLF5 and the tumorigenesis of breast cancer in vivo [38,40]. At the same time, another study showed that HDAC inhibitors partially expanded the number of BCSCs through the β-catenin signaling pathway [38,41].

### 3.2. Non-coding RNAs 

Many microRNAs (miRNAs) were reported to regulate BCSCs (Table 1). The miR-200c family was reported to inhibit BCSCs by targeting peptidylprolyl cis/trans isomerase, NIMA-interacting 1 (Pin1) [42]. It also suppresses the expression of BMI1 and the recombinant suppressor of zeste 12 homolog (Suz12) [42]. Additionally, the Let-7 family reduces the self-renewal capacity of BCSCs by inhibiting the Wnt signaling pathway [43]. In contrast, miRNA-221/222 promotes BCSC self-renewal by downregulating PTEN expression [44,45], and miR-20b-5p promotes the proliferation and inhibits the apoptosis of BCSCs [46].

Similarly, long noncoding RNAs (lncRNAs) also regulate BCSCs (Table 2). HOTAIR promotes the characteristics of BCSCs through regulating miR-34a/SRY-box transcription factor 2 (SOX2) [61]. SOX21-AS1 maintains BCSC stemness by enhancing the nuclear localization ability of the Yes-associated protein (YAP) [62]. Conversely, FGF13-AS1 inhibits the stemness of BCSCs, which destroys the interaction between insulin-like growth factor 2 mRNA-binding proteins (IGF2BPs) and MYC [63].

### 3.3. Transcription Factors and Signal Transduction Pathways

#### 3.3.1. Transcription Factors and Co-Activators

In addition to epigenetic factors, BCSCs are also regulated by transcription factors (Table 3). Octamer-binding transcription factor-4 (OCT4) plays a vital role in stem cell self-renewal. The high expression of OCT4 in 4T1 breast cancer cells enhances the mammosphere formation of CSCs in vitro [77]. The knockdown of OCT4 in an MCF-7 tumor-model induced apoptosis and inhibited tumor growth [78]. Estrogen induced OCT4 expression in MCF-7 cells and promoted mammosphere formation [79]. Programmed death ligand (PD-L1) maintains CSC stemness by promoting OCT4 phosphorylation, but the histone demethylase jumonji domain-containing protein-3 (JMJD3) decreases breast cancer stem cell-like properties by downregulating OCT4 [80,81].

KLF4 and KLF5 play oncogenic roles in breast tumors. KLF4 overexpression increases the proportion of CSCs [82] whereas miR-7 hinders the self-renewal and invasion abilities of CSCs by targeting KLF4 [83]. Lysine demethylase 7A (KDM7A) maintains BCSCs by upregulating KLF4 and c-MYC [84]. The downregulation of dual-specificity tyrosine phosphorylation-regulated kinase 2 (DYRK2) increases the expression of KLF4 and the proportion of BCSCs [85]. Our previous study found that KLF5 was essential for maintaining the stemness of normal breast stem cells and BCSCs in basal-like breast cancer (BLBC) [58,86]. PRMT5 increases the stemness of CSCs in BLBC by stabilizing KLF5 protein [37]. Mifepristone and metformin suppress BCSCs by inhibiting KLF5 expression [58,87]. Interestingly, several KLF5 downstream target genes, including SLUG [86,88] and NANOG [89,90], also increase the stemness of BCSCs.

C-MYC and SOX2 are two stem cell transcription factors. The overexpression of c-MYC in MDA-MB-468 cells facilitates the expression of ALDH [91]. Caveolin-1 inhibits the c-MYC-mediated metabolic reprogramming function of BCSCs [92]. In addition, p62 enhances the stem-like properties of BCSCs by stabilizing c-MYC [93]. SOX2 knockdown reduces BCSC stemness [94]. The tumor suppressor transcriptional repressor GATA binding 1 (TRPS1) inhibits SOX2 expression and the tumorigenic ability of CSCs [95]. Similarly, FOXO3a inhibits SOX2 expression and BCSC tumorigenicity [96]. Additionally, knockdown of SOX9 significantly inhibits the tumorigenicity of MDA-MB-231 cells, with a 70-fold decrease in tumor initiation capacity and a 40-fold increase in the ability to inhibit lung metastasis compared with the control [97]. More importantly, SOX9 and SLUG have significant advantages in the synergistic reversal of differentiated luminal cells into mammary stem cells [97].

#### 3.3.2. Signal Transduction Pathways

**Figure 1:**
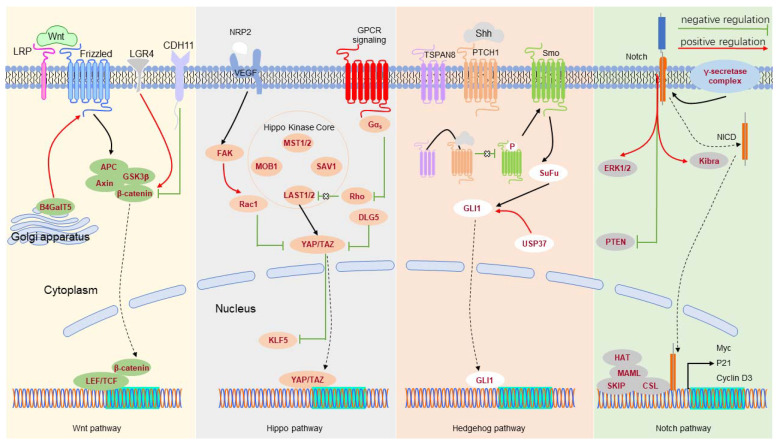
Critical signal transduction pathway networks associated with BCSCs.

The signal transduction pathways control gene expression in response to environmental stimuli (Table 4 and Figure 1). In TNBC, NOTCH-1 maintains CSC stemness, and inhibition of NOTCH-1 significantly reduces the number of CSCs [113,114]. Additionally, NOTCH-1 maintains the survival of BCSCs by inhibiting PTEN and activating extracellular signal-regulated kinase 1/2 (ERK1/2) [115]. NOTCH-2 knockdown can reduce the percentage of BCSCs [116]. NOTCH-3 inhibits the epithelial-to-mesenchymal transition (EMT) of BCSCs via the Kibra-mediated Hippo pathway [116], and inhibits the self-renewal of BCSCs by the interleukin-6 (IL6)/STAT3 signaling pathway [117]. NOTCH-4 plays a specific role in differentiating BCSCs into progenitor cells and reduces the expression level of NOTCH-4, impacting on its ability to form mammospheres [114].

Leucine-rich repeat-containing G protein-coupled receptor 4 (LGR4) acts as the primary positive regulator of the Wnt/β-catenin signaling pathway to maintain BCSCs [118]. CDH11 targets β-catenin, thereby inhibiting the stemness of TNBC cells [119]. Silencing LDL receptor-related protein 8 (LRP8) can reduce the percentage of BCSCs in TNBC [120]. β1,4-Galactosyltransferase V (B4GalT5) stabilizes Frizzled-1 by glycosylation and maintains BCSC stemness [121].

The activation of the Hedgehog signaling pathway also increases the number of BCSCs and the formation of mammospheres. TSPAN8 is upregulated in BCSCs, interacts with the SHH-PTCH1 complex, and promotes CSC stemness [14]. TSPAN8 was recently found to enter the nucleus by binding to 14-3-3θ, importin-β, and cholesterol [14]. Ubiquitin-specific peptidase 37 (USP37) activates the Hedgehog pathway by increasing Smo and GLI1 expression levels to enhance the characteristics of BCSCs [122]. The GLI1-derived tumors amplify a portion of CSCs featured by Keratin 6 and BMI1, supporting the role of Hedgehog signaling in breast cancer development [123].

TAZ, an effector of the Hippo pathway, is necessary to maintain the self-renewal ability of BCSCs [124]. Vascular endothelial growth factor (VEGF)/neuropilin-2 (NRP2) signaling participates in TAZ activation through a Rac1-dependent mechanism, enhancing the sphere-forming ability of BCSCs [125]. Silencing of the discs large homolog 5 (DLG5) might improve the activity of TAZ, thereby maintaining the self-renewal ability and stemness of BSCSs [126]. We previously reported that TAZ can stabilize KLF5 [127] and that tumor necrosis factor-alpha (TNFɑ) induces TAZ expression to increase the stemness of BCSCs [128]. Similarly, YAP was reported to promote the stemness of BCSCs [129]. Activating the competing endogenous RNA (ceRNA) network associated with star-related lipid transfer domain-containing 13 (STARD13) reduces YAP/YAZ activity, thereby inhibiting the formation of BCSCs [130]. YAP interacts with β-catenin, and TEA domain transcription factor 4 (TEAD4) cooperates in the nucleus to modulate CSCs in BLBC [131].

Receptor tyrosine kinases (RTKs) can activate the downstream PI3K/AKT/mTOR and MAPK signaling pathways. Type 1 insulin-like growth factor receptor (IGF-1R) maintains BCSCs by activating the downstream PI3K/Akt/mTOR pathway [132]. The downregulation of hypoxia-inducible factor 2 alpha (HIF-2α) expression can inhibit BCSCs by inhibiting the PI3K/AKT/mTOR signaling pathway [133]. B7-H3 activates MEK by binding to major vault protein (MVP), which regulates the MAPK kinase pathway and increases the proportion of BCSCs [134]. EGFR upregulates cyclooxygenase 2 (COX-2) to activate Nodal signaling and promote BCSC self-renewal [135]. SGCE stabilizes the level of EGFR by breaking the interaction between c-Cbl and EGFR, activating the PI3K/Akt pathway to foster breast cell stemness [16].

Wang et al. reported that the JAK/STAT3 pathway promotes BCSC differentiation by regulating lipid metabolism and that inhibition of this pathway blocks BCSC self-renewal [136]. WW domain-containing oxidoreductase (WWOX) inhibits the proliferation and metastasis of breast cancer cells by inhibiting the phosphorylation of JAK2 to hinder STAT3 activation [137]. WWOX also inhibits the expression of KLF5 in breast cancer [138]. In contrast, in TNBC, EGFR activates the JAK/STAT3 pathway by promoting the phosphorylation of STAT3, thereby facilitating the proliferation and invasion of tumor cells [139].

Alec et al. found that transforming growth factor-beta (TGF-β) activated the cytokine receptor leukemia inhibitory factor-receptor (LIFR) to initiate the JAK-STAT signaling pathway, subsequently driving the formation of BCSCs [140]. Jun et al. showed that TGF-β enhances the self-renewal capacity of BCSCs by upregulating fibronectin and Smad3-dependent COX-2 expression [141].

The transcription factor NF-κB is overexpressed in BCSCs, and high NF-κB activity regulates the self-renewal and differentiation of BCSCs [142]. Interleukin-1 alpha (IL-1α) mediates the HER2-induced NF-κB pathway to maintain BCSCs [143]. TNF-α promotes the self-renewal of BCSCs in human breast cancer cell lines by upregulating TAZ expression through the atypical NF-κB pathway [128]. HGFL-RON signaling increases the self-renewal capacity of BCSCs by activating β-catenin and its effector NF-κB [144]. Heregulin (HRG), the ligand of Erb-B2 receptor tyrosine kinase 3 (ErbB3), has been proved to stimulate mammosphere formation, which is achieved by activating the PI3K/NF-κB pathway [145]. Heat shock protein 27 (Hsp27) degrades IκBα to activate NF-κB and maintain BCSCs [146].

The scaffold protein SH3 domain containing ring finger 3 (SH3RF3), which is upregulated in BCSCs, interacts with c-Jun N-terminal kinases (JNK) in a JNK-interacting protein (JIP)-dependent manner and phosphorylates the latter, thereby activating the JNK-JUN pathway. This promotes the characteristics of BCSCs by enhancing the expression of pentraxin 3 (PTX3) [147].

## 4. Conclusions

Although the 5-year survival rate of breast cancer has been dramatically improved, recurrence is still a big challenge for breast cancer treatment. BCSCs are closely associated with recurrence. Notwithstanding that biomarkers have demonstrated their value in identifying BCSCs, it is still difficult to distinguish between normal stem cells and BCSCs. Due to the heterogeneity of breast cancer, the expressions of BCSC markers are varied and show distinct clinical values in different breast cancer subtypes. Moreover, distinct BCSC subclones may co-exist with a heterogeneous tumor, and new BCSC subclones may be induced during tumor treatment. Therefore, it is necessary to combine the analysis of BCSC markers in certain phases during tumor progression to improve BCSC-based prognosis. The continuous updating of breast cancer-specific biomarkers has an important guiding significance for CSC identification and separation. Novel therapeutic strategies that target BCSC and BCSC niches and the rest of the cancer are required to target the entire cancer and prevent metastasis or recurrence. Currently, there are several treatments for BCSCs, including cytotherapy (NCT02915445), antibody-based biopharmaceuticals (NCT01954355), synthetic small molecule compounds (NCT00645333), natural compounds and their products (NCT01608867), and nucleic acid medicines. In the I-SPY2 (Investigation of serial studies to predict your therapeutic response with imaging and molecular analysis) clinical trial platform, 17 new agents were evaluated in combination with neoadjuvant chemotherapy for women with locally advanced breast cancer [148]. By conducting such clinical trials, promising drugs were approved more quickly, and the drug development process was shortened [149].

Cancer occurrence is a multi-factor, multi-stage, multi-gene mutation accumulation process. Various signaling pathways may be involved in cancer occurrence and development simultaneously. Most of the signaling pathways regulating BCSCs are evolutionarily conserved and shared with normal stem cells, which makes them inappropriate as therapeutic targets. With a deep understanding of BCSCs, we expect more specific regulation of BCSCs pathway factors to be found. The relationship between multiple pathways is closely supported, providing a more theoretical basis for developing new targeted therapies in order to overcome current breast cancer treatment limitations.

## Figures and Tables

**Table 1:** miRNAs regulating BCSCs.

Names	Mechanism	References
miRNA-200c	inhibits the expression of Pin1, BMI1 and Suz12.	[42]
Let-7 family	inhibits the Wnt signaling pathway.	[43]
miR-34 family	targets Notch1.	[47]
miRNA-146a	promotes the asymmetric division of BCSCs.	[48]
miRNA-760	inhibits the expression of NANOG.	[49]
miR-422a	reduces the expression of Proteolipid Protein 2 (PLP2).	[50]
miRNA-142-3p	targets β-catenin.	[51]
miRNA-1	targets ecotropic virus integration site 1 (EVI1).	[52]
miRNA-128-3p	downregulates NIMA related kinase 2 (NEK2) to inhibit the Wnt signaling pathway.	[53]
miRNA-638	reduces the expression of E2F2.	[54]
miR-376c-3p	reduces the expression of RAB2A.	[55]
miRNA-221/222	inhibits PTEN expression.	[44]
miR-20b-5p	bidirectionally regulates cyclin D1 and E2F1.	[46]
miR-335	inhibits cadherin 11 (CDH11), β-catenin, and vimentin.	[56]
miR-153	downregulates hypoxia-inducible factor 1 subunit alpha (HIF1ɑ) and KLF5.	[57,58]
miR-145	suppresses BCSCs growth by inhibiting KLF4.	[59]
miR-375	decreases BCSCs by interrupting the JAK2-STAT3 pathway.	[60]

**Table 2:** LncRNAs regulating BCSCs.

Names	Mechanism	References
HOTAIR	regulates miR-34a to upregulate the expression of SOX2 in BCSCs.	[61]
SOX21-AS1	inhibits the Hippo signaling pathway.	[62]
CCAT1	enhances the expression of T-cell factor 4 (TCF4) to activate the Wnt signaling pathway.	[64]
H19	forms a two-way negative feedback loop with miRNA let-7 and LIN28.	[65]
SPRY4-IT1	sponges miR-6882-3p.	[66]
LINC00511	regulates the miR-185-3p/E2F1/NANOG axis.	[67]
HOTTIP	acts as an miR-148a-3p sponge and regulates Wnt 1.	[68]
LUCAT1	competitively binds to miR-5582-3p and transcription factor 7 like 2 (TCF7L2) to enhance the Wnt/β-catenin pathway.	[69]
FEZF1-AS1	regulates the miR-30a/NANOG signal pathway.	[70]
Lnc408	recruit specificity protein 3 (Sp3) to inhibit chibby family member 1 (CBY1) and β-catenin expression.	[71]
CCAT2	upregulates OCT4-PG1 and the miR-205-Notch1 pathway.	[72]
Hh	stimulates hedgehog signaling.	[73]
Lnc030	interacts with poly (RC) binding protein 2 (PCBP2) to stabilize squalene epoxidase (SQLE) and activate the PI3K/Akt signaling pathway.	[74]
MALAT1	positively regulates SOX2.	[75]
ROPM	maintains group XVI phospholipase A2 (PLA2G16) to facilitate lipid metabolism, thereby activating the Wnt/β-catenin pathway.	[76]
FGF13-AS1	regulates the IGF2BPs/Myc feedback loop.	[63]

**Table 3:** Transcription factors and co-activators regulating BCSCs.

Names	Hallmarks	References
OCT4	OCT4 promotes sphere formation of BCSCs in vitro, while inhibition of OCT4 induces apoptosis, reduces BCSC characteristics, and inhibits tumor growth.	[77,78,79]
KLF4	KDM7A and DYRK2 increase BCSCs by upregulating KLF4 expression, and miR-7 inhibits KLF4 to inhibit BCSCs self-renewal and invasion.	[83,84,85]
KLF5	Mifepristone and metformin inhibit KLF5 and BCSC. PRMT5 increases stemness of BCSC by stabilizing KLF5.	[37,58,87]
C-MYC	Caveoli-1 inhibits C-MYC-mediated BSCS metabolic reprogramming and p62 stabilizes C-MYC to enhance BCSC properties.	[92,93]
SOX2	Knockdown of SOX2 attenuates stemness of BCSC. TRPS1 and FOXO3a inhibit SOX2 the expression and tumorigenesis of BCSCs.	[94,95,96]
SOX9	Knockdown of SOX9 significantly inhibits the tumorigenicity of MDA-MB-231 cells.	[97]
SLUG	Hes family BHLH transcription factor 1 (HES1) increases SLUG transcription and BCSC stemness. Interestingly, the Notch4/SLUG/Gas1 axis maintains mesenchymal-like BCSCs.	[98,99]
SNAIL	Uncoupling Protein 1 (UCP1)-mediated fructose-bisphosphatase 1 (FBP1) expression promotes BCSC properties, which can be reversed by SNAIL. Interferon beta (IFN-β restrains SNAIL-induced tumor initiation.	[100,101]
β-catenin	β-catenin facilitates BCSC properties through CCL2-mediated macrophage polarization and infiltration. CCL16 and mortalin maintain the stemness of BCSCs by promoting the translocation of β-catenin.	[102,103,104]
GLI1	Tripartite motif 16 (TRIM16) inhibits BCSCs partially via Glioma-related homologue 1 (GLI1). In contrast, estrogen promotes BCSCs by activating GLI1.	[105,106]
p65	p65 is important for BCSC survival.	[107]
ERα	Reduction in expression of ER inhibits CSC tumor-seeding efficiency.	[108]
FOXO3a	FOXO3a inhibits the characteristics and tumorigenesis of BCSCs by negatively regulating SOX2.	[96]
TAZ	Overexpression of transcription activator with PDZ-binding motif (TAZ) in BCSCs enhances tumorigenicity and cell migration. The ability of HIF1 and Crumbs homolog 3 (CRB3) to maintain or induce BCSC properties is partially achieved by activating TAZ.	[109,110,111]
YAP	Downregulation of YAP1 has a negative effect on BCSC tumorigenicity and stemness markers.	[112]

**Table 4:** Signal transduction pathways regulating BCSCs.

Related Signaling Pathway Factors		Mechanism	References
NOTCH	NOTCH-1	inhibits PTEN and activates ERK1/2 to maintain BCSCs.	[115]
	NOTCH-2	promotes BCSC survival.	[116]
	NOTCH-3	inhibits BCSC self-renewal by IL6/STAT3.	[117]
	NOTCH-4	promotes mammosphere formation.	[114]
WNT	LGR4	promotes BCSCs.	[118]
	CDH11	inhibits TNBC cell stemness.	[119]
	LRP8	decreases the percentage of BCSCs.	[120]
	B4GalT5	maintains BCSCs by stabilizing Frizzled.	[121]
HH	TSPAN8	interacts with the SHH-PTCH1 complex to promote stemness of breast cancer.	[14]
	USP37	increases Smo and GLI1 expression to enhance BCSC characteristics.	[122]
HIPPO	VEGF/NRP2	activates TAZ to enhance BCSC sphere-forming ability.	[125]
	DLG5	enhances TAZ activity to maintain BCSC self-renewal.	[126]
	TNF-ɑ	induces TAZ expression to increase BCSC stemness.	[127,128]
	STARD13	reduces YAP/YAZ activity, thereby inhibiting the formation of BCSCs.	[130]
RTK	IGF-1R	maintains BCSCs by activating the PI3K/Akt/mTOR pathway.	[132]
	HIF-2ɑ	inhibits BCSCs by inhibiting the PI3K/Akt/mTOR pathway.	[133]
	B7-H3	activates MEK and increases BCSC proportions.	[134]
	SGCE	stabilizes EGFR levels, thereby fostering breast cell stemness.	[16]
JAK/STAT3	WWOX	hinders STAT3 activation to block breast cancer cell proliferation and metastasis.	[137]
	EGFR	promotes STAT3 phosphorylation to facilitate tumor cell proliferation and invasion.	[139]
TGF-β	LIFR	drives the formation of BCSCs.	[140]
	Fibronectin, COX2	enhances the self-renewal capacity of BCSCs.	[141]
NF-κB	IL-1α	maintains BCSCs.	[143]
	HGFL-RON	supports the self-renewal capacity of BCSCs.	[144]
	HRG	stimulates mammosphere formation.	[145]
	Hsp27	degrades IκBα to maintain BCSCs.	[146]

## Data Availability

Not applicable.
